# Surgery for Functional Tricuspid Regurgitation in Adult Atrial Septal Defect — An Increasing Subject in a Decreasing Matter

**DOI:** 10.21470/1678-9741-2020-0503

**Published:** 2022

**Authors:** Andrei George Iosifescu, Alexandru Popescu, Toma Andrei Iosifescu, Alina Teodora Timişescu, Sorin Maximeasa, Vlad Anton Iliescu

**Affiliations:** 1 Faculty of Medicine, University of Medicine and Pharmacy “Carol Davila”, Bucharest, Romania.; 2 Department of Cardiac Surgery, Emergency Institute for Cardiovascular Diseases “Prof. Dr. C.C. Iliescu”, Bucharest, Romania.; 3 Department of Cardiac Surgery, Monza Hospital, Bucharest, Romania.

**Keywords:** Atrial Heart Septal Defects, Tricuspid Valve Insufficiency, Adult, Cardiac Surgical Procedures, Decision Making

## Abstract

**Introduction:**

Functional tricuspid regurgitation (TR) is known to complicate adult atrial septal defect (ASD), but its management is still under debate. We reviewed our experience in ASD surgery, focusing on associated functional TR and its treatment.

**Methods:**

This retrospective study (2005-2019) included 206 consecutive adult ASD surgical cases without associated valve pathology, except functional TR. Variables were statistically compared on TR classes and surgery-defined groups.

**Results:**

Mean age of the patients was 40.3±13 years; 19.9% had sinus venosus syndrome. TR severity was directly related to age, pulmonary systolic pressure, right ventricular and tricuspid annulus diameters, and heart failure class. TR ≥ 2 was found in 134 (65%) patients, while TR ≥ 3 in 56 (27.2%) patients. Tricuspid surgery was associated to shunt closure in 66 (32%) patients, almost all through valve repair; indication was directly related to age, right ventricular and tricuspid annulus diameters, and heart failure class ≥ 3. Tricuspid surgery was more efficient than isolated shunt closure in decreasing TR (79±23% vs. 36±26%; P=1.8 E-18). Device closure availability (last four years of the study) was associated with 1/3 reduction of surgical cases but increased the share of cases with TR>2 (> 51% vs. < 31%; P<0.05).

**Conclusion:**

In the era of device closure, surgery for adult ASD is less frequent, but the share of significant TR cases is in net increase. To avoid long-term postoperative TR, we plead for valve repair in all patients with severe TR and for considering repair in moderate TR at risk of persistence.

**Table:** Abbreviations, Acronyms & Symbols

%ΔTR	= Percentage of TR decrease	PAPVC	= Partial anomalous pulmonary venous connection
ΔRVEDD	= RVEDD variation	PASP	= Pulmonary artery systolic pressure
ΔTR	= TR variation	Qp/Qs	= Ratio between pulmonary and systemic output
AF	= Atrial fibrillation	RV	= Right ventricular
ANOVA	= Analysis of variance	RVEDD	= Right ventricular end-diastolic diameter
ASD	= Atrial septal defect	TAD	= Tricuspid annulus diameter
CAGB	= Coronary artery bypass grafting	TR	= Tricuspid regurgitation
LVEF	= Left ventricular ejection fraction	TV	= Tricuspid valve
NYHA	= New York Heart Association	TVR	= Tricuspid valve repair
PA	= Pulmonary artery		

## INTRODUCTION

Atrial septal defect (ASD) in adult patients is frequently complicated by functional tricuspid regurgitation (TR) due to right ventricular (RV) remodeling and tricuspid annulus dilation secondary to chronic RV volume overload. While for the management of significant functional TR associated with left heart disease there is a large consensus regarding simultaneous tricuspid valve repair (TVR) at the time of left-sided surgery, attitude toward TR associated with ASD is still under debate.

In the current era, adult patients with ASD are subject to interventional device closure or to surgical repair, both with good reported results. The respective populations can hardly be considered similar as interventional treatment is not applied to patients with defects “too large for device”, nor in those without adequate rim, neither in cases with associated partial anomalous pulmonary venous connection (PAPVC) or in atrioventricular septal defects; furthermore, there is a potential referral bias toward surgery in patients with severe TR and extreme right heart dilation^[1]^.

The objectives of this study are to review our experience in the surgical repair of ASD and/or PAPVC, to outline the results and indications of surgery for functional TR in the management of ASD, and to study the impact of interventional closure of ASD on the surgical practice for the disease.

## METHODS

This retrospective study included 206 consecutive cases of adult (≥ 18 years old) ASD and/or PAPVC, operated between 2005 and 2019; as we studied the management of functional TR secondary to supraventricular shunts, patients with associated indication of mitral valve surgery (including atrioventricular septal defect), with Ebstein disease, or with significant aortic or pulmonary valve disease were excluded; redo surgical cases were also not included.

At admission, each patient gave his/her consent for the use of his/her medical data for scientific purposes. The study was approved by the Institutional Ethics Committee of our hospital (No.32194/2019).

Data collection was performed on a Microsoft Excel 2010 database. For each patient, the following variables were recorded: age, sex, type of disease (ASD/PAPVC), operation date, surgical technique, New York Heart Association (NYHA) heart failure class at admission, preoperative associated disorders (atrial fibrillation, coronary disease), name of the surgeon, transthoracic echocardiographic data (ASD diameter, preoperative and postoperative RV end-diastolic diameter [RVEDD] measured in apical four-chamber view, tricuspid annulus diameter [TAD], preoperative and at discharge degree of TR, left ventricular ejection fraction [LVEF], and estimated pulmonary artery systolic pressure [PASP]), the ratio between pulmonary and systemic output (if available), postoperative events (in-hospital complications, death), and length of in-hospital stay.

Through two-dimensional and Doppler echocardiography, TR was graded qualitatively and quantitatively as mild, moderate, severe, and massive (1 to 4, respectively), with vena contracta and effective regurgitant orifice area cutoff values between TR classes set at 3, 7, and 13 mm and 20, 40, and 60 mm^2^, respectively; intermediate values found in collected data were noted accordingly (TR 2-3 = 2.5). Differences between preoperative and postoperative values of TR grade and of RVEDD were calculated for each case and named “TR variation” (∆TR) and “RVEDD variation”, respectively. Percentage expression of ∆TR from preoperative TR grade was noted as %∆TR (percentage of TR decrease).

We compared clinical data and operative results on subgroups defined by:

- Different degrees of TR (no/mild, moderate, more than moderate).
- In cases with preoperative TR ≥ 2, we compared patients with additional tricuspid valve (TV) surgery to those with shunt closure only.
- Patients operated on between 2005 and 2015 (when device closure was sporadic) were compared to those treated after 2016 (when percutaneous closure became available).

Clinical characteristics and echocardiographic parameters were compared using the Student’s *t*-test, Mann-Whitney U test, and one-way analysis of variance for continuous variables, and χ^2^ test (2×2 or 3×2) for dichotomous variables. Calculation was performed using Social Science Statistics online calculator (www.socscistatistics.com) and the embedded statistical functions in Microsoft Excel.

## RESULTS

The study group included 206 cases, mean age 40.3±13 years (extremes 18 and 76 years), and it showed female sex dominance (138/68 ≈ 2:1). One hundred sixty-five of these patients had isolated ASD, and 41 had PAPVC. In the latter group, the anomalous pulmonary veins drained to the superior vena cava in 36 patients (87.8%) and to the right atrium in all other cases. At admission, most patients (71.8%) were in NYHA class II, 32 (15.5%) had a history of atrial fibrillation, six had coronary artery disease requiring revascularization (≈ 3%), and two had pulmonary artery aneurysm with surgical indication (≈ 1%). All operations were performed through median sternotomy, with cardiopulmonary bypass, using cold hyperkalemic blood cardioplegia. The ASD was closed with a pericardial patch in 97.6% (n=201) of the cases. The pericardial baffle technique was employed for PAPVC repair; patch enlargement of the superior cavoatrial junction was associated in 36 (87.8%) of PAPVC cases.

Concomitant TV surgery was performed in 66 patients (32%), consisting almost entirely of TVR (65 cases). Rigid ring annuloplasty (Edwards-MC3®, Carpentier-Edwards Physio® and Classic®) was the preferred technique (n=36); flexible rings (n=8), DeVega procedure (n=9), and Kay repair (n=12) were also employed. In severe TV tethering, pericardial patch augmentation of the anterior cusp was performed (n=3). Operations were done by 12 different surgeons, with an unequal burden of cases (1% to 50%). The decision to perform TVR was left to the surgeon.

Statistical data of the whole adult ASD/PAPVC population is shown in Table 1. The mean preoperative TR grade was 2±1, and TV surgery was performed in 32% of the patients. Postoperative results were good; 37 (18%) patients had complications (mostly of benign nature); half of them were rhythm disorders. There was one in-hospital death caused by biventricular failure followed by multiple organ failure in a 39-year-old male with a history of severe alcohol addiction.

**Table 1:** General clinical data.

Number of patients	206
Age (years)	40.3±13
Female:Male	138:68 (≈2)
PAPVC	41 (19.9%)
NYHA heart failure class	
I	10 (4.9%)
II	147 (71.4%)
III	47 (22.8%)
IV	2 (0.9%)
Preoperative AF	32 (15.5%)
ASD diameter (mm)	24.2±8.1
Preoperative TR grade (0-4)	2±1
TR surgery	66 (32%)
Other associated operations:	
CABG	6 (≈3%)
Treatment of pulmonary artery aneurysm	2 (≈1%)
Preoperative RVEDD (mm)	46.1±7.7
Preoperative TAD (mm)	38.9±5.1
Preoperative LVEF (mm)	57.4±6.2
Preoperative PASP (mmHg)	44.3±11
Preoperative Qp/Qs	2.55±0.8
Postoperative RVEDD (mm)	39.1±7
ΔRVEDD	7.6±5.9
Postoperative TR grade (0-4)	0.85±0.7
ΔTR	1.22±1
Complications	37 (18%)
Death	1 (0.49%)
Hospital stay (days)	8.8 ±3.3

ΔRVEDD=RVEDD variation; ΔTR=TR variation; AF=atrial fibrillation; ASD=atrial septal defect; CABG=coronary artery bypass grafting; LVEF=left ventricular ejection fraction; NYHA=New York Heart Association; PAPVC=partial anomalous pulmonary venous connection; PASP=pulmonary artery systolic pressure; Qp/Qs=ratio between pulmonary and systemic output; RVEDD=right ventricular end-diastolic diameter; TAD=tricuspid annulus diameter; TR=tricuspid regurgitation

Behavior of the studied variables was compared in subgroups defined by TR degree (no/mild, moderate, and more than moderate TR) (see Table 2); in only 6.3% of the patients, there was no TR. TR severity had a significant direct relation to age, PASP, TAD, and RVEDD; it was also significantly related to NYHA class ≥ III and to lower LVEF — which is probably explained by ventricular interaction in the context of RV dilation.

**Table 2:** Comparison of tricuspid regurgitation (TR) class defined subgroups.

	TR < 2 (No/mild)	TR = 2 (Moderate)	TR > 2 (> Moderate)	*P*-value (ANOVA, 3×2 χ^2^-test)
Number (%)	72 (≈35%)	62 (≈30%)	72 (≈35%)	
Age (years)	35.7±12.6	39.5±11.7	45.6±12.4	*P*<0.00005
Female:Male	53:19 (≈2.8)	38:24 (≈1.6)	47:25 (≈1.8)	NS
PAPVC	13 (18.1%)	11 (17.7%)	17 (23.6%)	NS
NYHA heart failure class ≥ III	11 (15.3%)	10 (16.1%)	28 (38.9%)	*P*<0.001
Preoperative AF	9 (12.5%)	9 (14.5%)	14 (19.4%)	NS
ASD diameter (mm)	22.4±6.8	25.4±8.5	25±8.6	*P*=0.07
Preoperative TR grade (0-4)	0.93±0.48	2	3.05±0.45	
TR surgery	0	12 (16.7%)	54 (75%)	
Preoperative RVEDD (mm)	42.8±6.8	46.4±7.7	49.2±7.3	*P*<5 E-6
Preoperative TAD (mm)	36.1±.4.8	38.7±4.6	41.9±4.1	*P*=1.6 E-11
Preoperative LVEF (mm)	59.7±6.4	56.7±5.9	55.7±5.4	*P*<0.0005
Preoperative PASP (mmHg)	40.5±10.6	43.8±10.8	57.5±10.4	*P*<0.005
Preoperative Qp/Qs	2.4±0.9	2.7±0.6	2.6±0.6	NS
Postoperative RVEDD (mm)	36.3±5.5	39.5±7.5	41±7	*P*<0.005
ΔRVEDD	6.9±6.2	7.3±5.9	8.5±5.7	NS
Postoperative TR grade (0-4)	0.53±0.5	1.10 ±0.68	0.98±0.81	
ΔTR	0.48±0.45	0.92±0.68	2.06±0.95	<1.15 E-25
Complications	12 (16.7%)	10 (16.3%)	15 (20.8%)	NS
Death	0	0	1 (1.38%)	NS
Hospital stay (days)	8.5±3.5	8.8±2.4	9.1±3.6	NS

ΔRVEDD=RVEDD variation; ΔTR=TR variation; AF=atrial fibrillation; ANOVA=analysis of variance; ASD=atrial septal defect; LVEF=left ventricular ejection fraction; NYHA=New York Heart Association; PAPVC=partial anomalous pulmonary venous connection; PASP=pulmonary artery systolic pressure; Qp/Qs=ratio between pulmonary and systemic output; RVEDD=right ventricular end-diastolic diameter; TAD=tricuspid annulus diameter

We performed TV surgery in 32% of our ASD/PAPVC adult population. It was indicated in all patients with TR grade > 3 (n=13), in 31 of 43 (72.1%) patients with TR grade = 3, 10 of 16 (62.5%) patients with TR grade = 2.5, in 12 of 62 (19.4%) patients with TR grade = 2, and in none of the 72 patients with TR grade < 2.

We further focused on the 134 patients with TR ≥ 2, in which we compared the cases with tricuspid surgery (n=66) to those treated only by shunt closure (n=68) (see Table 3); we have not included in this comparison patients with no/mild TR in which the decision to refrain from TVR was indisputable.

**Table 3:** Patients with tricuspid regurgitation (TR) ≥ 2: shunt closure only group *vs.* additional tricuspid valve surgery group.

	Shunt closure only (n=68)	Tricuspid valve surgery (n=66)	*P*-value (*t*-test, χ^2^test)
Age (years)	40.1±11.5	45.5±12.9	0.011
Sex (Female:Male)	44:24:00	41:25:00	NS
PAPVC	12 (17.6%)	16 (24.2%)	NS
NYHA heart failure class ≥ III	13 (19.1%)	25 (37.8%)	*P*=0.016
Preoperative AF (active/history)	11 (16.2%)	12 (18.2%)	NS
ASD diameter (mm)	24.2±8	26.2±9	NS
Preoperative TR grade (0-4)	2.22±0.4	2.92±0.6	4.5E-12
Preoperative RVEDD (mm)	45.6±6.1	50.2±8.3	*P*<0.0005
Preoperative TAD (mm)	37.3±4.5	42.2±4.7	1.5E-10
Preoperative LVEF (mm)	56.7±6	55.6±5.3	NS
Preoperative PASP (mmHg)	44.9±11	46.8±10.4	NS
Preoperative Qp/Qs	2.6±0.6	2.7±0.7	NS
Postoperative RVEDD (mm)	38.5±5	42.3 ±8.3	*P*<0.01
ΔRVEDD	7.1±5.8	8.8±5.7	NS
Postoperative TR grade (0-4)	1.42±0.6	0.62±0.68	5.7E-11
ΔTR	0.8±0.55	2.3±0.77	6.8E-24
%ΔTR	36±26%	79±23%	
Complications	12 (17.6%)	13 (19.7%)	NS
Death	0	1 (1.5%)	NS
Hospital stay (days)	8.7±2.5	9.2±3.7	NS

%ΔTR=percentage of TR decrease; ΔRVEDD=RVEDD variation; ΔTR=TR variation; AF=atrial fibrillation; ASD=atrial septal defect; LVEF=left ventricular ejection fraction; NYHA=New York Heart Association; PAPVC=partial anomalous pulmonary venous connection; PASP=pulmonary artery systolic pressure; Qp/Qs=ratio between pulmonary and systemic output; RVEDD=right ventricular end-diastolic diameter; TAD=tricuspid annulus diameter

TV surgery was applied to significantly older patients, more frequently in NYHA class ≥ III, with larger preoperative RVEDD and TAD. TV surgery had a highly significant impact on TR grade, decreasing it from 2.92±0.6 (preoperatively) to 0.62±0.68 (prior to hospital discharge), *i.e.*, almost 80% of the initial value (79±23%). Patients with isolated ASD closure had also a decrease in TR grade (from 2.22±0.4 to 1.42±0.6), but its magnitude was only 36±26% of the preoperative value. TV surgery was highly more effective than shunt closure alone in decreasing functional TR (≥ 2/4) (*P*=1.8 E-18).

We compared the ∆TR recorded in patients with TV surgery or with shunt closure only on subgroups defined by preoperative TR grade (Table 4). In patients with TR ≥ 2, %∆TR was not related to the level of preoperative TR; patients treated through isolated shunt closure with TR = 2 had almost the same %∆TR as those with TR > 2 (≈ 36%), while in those subject to TV surgery, the magnitude was non-significantly different (Mann-Whitney U test). Both in patients with TR = 2 or TR > 2, TV surgery brought a more than double %∆TR compared to shunt closure alone.

**Table 4:** Nominal and percentage tricuspid regurgitation (TR) decrease after shunt closure, with and without tricuspid valve (TV) surgery, in different classes of TR.

Preoperative TR	TV surgery	N	Preoperative TR	Postoperative TR	∆TR	%∆TR
0 < TR < 2	No	59	1.14±0.2	0.65±0.47	0.48±0.45	43±42%
TR = 2	No	50	2	1.28±0.6	0.72±0.6	36±28.8%
	Yes	12	2	0.25±0.38	1.75±0.4	87.5±18%
TR > 2	No	18	2.83±0.24	1.81±0.48	1.03±0.4	36.7±14%
	Yes	54	3.12±0.48	0.7±0.7	2.42±0.8	77.4±23%

%ΔTR=percentage of TR decrease; ΔTR=TR variation

TR grade at discharge was ≥ 2 in 29 patients; most of them had not undergone TV surgery (n=24; 82.8%). Without TVR, the risk of moderate or greater TR at discharge was 26% (13/50) in patients with preoperative TR = 2 and 55.5% (10/18) in those with preoperative TR > 2 (*P*<0.05); with TV surgery, the same risks were 0 and 9.3% (5/54), respectively.

Trying to evaluate the impact of ASD device closure on surgical practice, we compared patients operated on before 2016 (when percutaneous treatment became a current procedure) to those surgically treated after this date (Table 5). As expected, we found a significant decrease in the burden of surgical cases/year (to approximatively 2/3 of the pre-device value). The patients’ clinical profile changed in the period of device-closure availability; surgical patients were older, had larger ASD diameter (28.4 mm *vs.* 23.2 mm), higher TR grade (2.4±1.1 *vs.* 1.9±0.9), larger preoperative RVEDD and TAD, and lower LVEF. TR ≥ 2 was more frequently found at operation in the post-device period (51.2%), compared to the former time (30.9%) (*P*<0.05); consequently, a significantly higher share of cases became subject to TV surgery (61% *vs.* 24.8%) (χ^2^; *P*<0.00001).

**Table 5:** Comparison of subgroups defined by atrial septal defect (ASD) device closure availability in our institution.

	2005-2015	2016-2019	*P*-value (*t*-test, χ^2^test)
ASD device closure available in hospital	No	Yes	
Number of patients	165	41	
Cases/year	15±4.3	10.3±1.8	*P*<0.05
Age (years)	39±12.2	45.5±14.5	*P*=0.012
PAPVC	33 (20%)	8 (19.5%)	NS
ASD diameter	23.2±7.4	28.4±9.2	*P*<0.005
Preoperative NYHA heart failure class ≥ III	33 (20%)	10 (24.3%)	NS
Atrial fibrillation	25 (15.2%)	7 (17.1%)	NS
Preoperative TR grade	1.9±0.9	2.4±1.1	*P*<0.01
Preoperative TAD	38.2±4.9	41.7±5.1	*P*<0.0005
Preoperative RVEDD	45.1±7.1	50.1±8.4	*P*=0.001
Preoperative PASP	44.6±10.8	42.9±11.5	NS
LVEF	58.1±6.3	54.6±4.6	*P*<0.0005
Qp/Qs	2.5±0.7	2.78±0.9	NS
Preoperative TR > 2	51 (30.9%)	21 (51.2%)	*P*<0.05
Patients with TV surgery: n (%)	41 (24.8%)	25 (61%)	*P*<0.00001
Postoperative TR grade	0.87±0.74	0.80±0.60	NS
Associated surgery (CABG, PA aneurysm)	2	6	*P*<0.05
Complicated cases: n (%)	27 (16.4%)	10 (24.4%)	NS
In-hospital death	1	0	NS
Hospital stay (days)	8.6±2.3	9.6±5.7	NS

CABG=coronary artery bypass grafting; LVEF=left ventricular ejection fraction; NYHA=New York Heart Association; PA=pulmonary artery; PAPVC=partial anomalous pulmonary venous connection; PASP=pulmonary artery systolic pressure; Qp/Qs=ratio between pulmonary and systemic output; RVEDD=right ventricular end-diastolic diameter; TAD=tricuspid annulus diameter; TR=tricuspid regurgitation; TV=tricuspid valve

## DISCUSSION

Functional TR due to tricuspid annulus dilation and RV remodeling secondary to long-standing right heart volume overload is a well-known complication in the natural history of adult ASD; it further contributes to heart failure aggravation. Incidence and severity of functional TR, in these patients, increased with age and pulmonary hypertension^[2]^.

TR is frequently associated with adult ASD, this is proven by the fact that almost all (93.7%) of our patients had some degree of TR: mild or less (< 2), moderate (= 2), and more than moderate (> 2); size of these classes amounted to approximately 35%, 30%, and 35%, respectively; another study had found TR ≥ 2 in adult ASD even more frequently (75%^[3]^, instead of the 65% noted by us).

Today we are facing an increasing proportion of patients with significant functional TR among surgically treated adult ASD. This may be due to an increase in the surgical referral of patients who were denied device closure (including the “too large for device” cases) and also of those with severe functional TR^[1]^. In the overall group, we found 35% with more than moderate TR (> 2); this proportion had increased over time — from 30.9%, when device closure was not a current option, to 51.2%, in the device closure era. Consequently, the incidence of TV surgery associated to adult ASD repair also witnessed a sharp increase after the introduction of device closure (up to 61%, in our study).

While in the current era the ratio of patients with significant TR among ASD/PAPVC surgical cases may exceed 50%, the management of functional TR associated with adult supraventricular shunt cases is still under debate. As specific guidelines on the subject are lacking, surgical decision is mainly based on (sometimes contradictory) information from three types of studies: a) extrapolation of abundant evidence concerning functional TR associated with left-sided heart disease; b) a few surgical studies on TR occurring in adult congenital heart disease with RV volume overload (ASD, PAPVC, or long-standing pulmonary regurgitation); and c) studies of residual TR after percutaneous closure of ASD.

In functional TR complicating left-sided heart disease, there is clear recommendation in favor of TVR simultaneous to left heart surgery: in severe TR (Class I), in mild to moderate TR with TAD ≥ 40 mm (or ≥ 21 mm/m^2^) (Class IIa), and in mild/moderate TR with recent right heart failure (Class IIb)^[4-7]^. Some authors extend these criteria for TVR in functional TR to any case of simultaneous cardiac surgery, thus, to ASD/PAPVC, as well^[8]^. Only recently the American College of Cardiology/American Heart Association 2018 Guidelines included a statement concerning this matter: “Concomitant tricuspid annuloplasty can be of benefit in patients with moderate or more TR, as the additional volume load may adversely affect RV remodeling”^[9]^.

Even if there are pathogenic differences between functional TR occurring in left heart disease and that met in supraventricular shunts, many surgeons (including most of our group) applied a similar attitude in both settings. This approach has been described by various authors. Jeong^[10]^ used the same TVR indications for mitral valve and ASD-related functional TR (TR ≥ 1 with annular dilatation or PASP > 50 mmHg with annular dilatation, irrespective of the TR grade). Similarly, Giamberti et al.^[11]^ reported associated TV surgery in 11% of their adult ASD population; they recommend TVR in all ASD with moderate or severe TR and also when TAD > 40 mm regardless the degree of TR (as Dreyfus suggested for functional TR associated with mitral valve disease^[12]^). Another group performed TVR only in cases with TR ≥ 3 (22.9%)^[13]^.

On the other hand, some studies have shown that isolated ASD closure promotes the reverse remodeling of the right heart and reduces the magnitude of associated TR^[2]^. The beneficial effect of defect closure would continue during the first year after the procedure; with this argument, some plead for transcatheter closure in cases with severe TR^[14,15]^ and others advocate against TVR even in surgical cases of ASD with severe TR^[16]^ (attitude to which our results firmly oppose).

However, most studies noted that a significant part of the patients with moderate or severe TR remain with persistent TR after transcatheter ASD closure: 30%^[14]^ to 50%^[2,17]^, or even 56%^[18]^. In our 68 patients with preoperative TR ≥ 2 treated without TV surgery (a group consisting mostly of cases with moderate regurgitation: mean preoperative TR = 2.2±0.4), we found persistence of TR ≥ 2 in 23 cases (33.8%). In our isolated closure patients, a preoperative TR > 2 was associated with a more than double risk of a postoperative TR ≥ 2, when compared to the preoperative TR = 2 cases. This pleads for systematic TVR in cases with preoperative TR > 2.

Various factors have been shown to be associated with TR persistence after ASD closure without TVR: increased age and PASP > 60 mmHg^[2,19]^; larger right ventricle, more severe preoperative TR, larger TAD and tenting area (TAD > 35 mm and tricuspid septal leaflet angle > 30° being highly predictive of persistent TR)^[17]^; permanent atrial fibrillation^[14]^; age ≥ 60 years, right atrial end-diastolic area ≥ 10 cm2/m2, RV systolic pressure ≥ 44 mmHg, and tricuspid annular plane systolic excursion ≤ 2.3 cm^[18]^. Consequently, the medical decision should lean toward performing TVR not only in cases with massive or severe TR (TR ≥ 3), but also in moderate-severe cases (3 > TR ≥ 2) proven to have some of the abovementioned risk factors.

The effectiveness of TVR associated with ASD closure is proven by the 79±23% reduction of TR grade found in our TV surgery group, while isolated ASD closure patients showed only a 36±26% TR grade reduction. It is also interesting that the percentage of decrease of TR in isolated closures was almost the same in patients with moderate and more than moderate TR.

Late TR worsening after mitral surgery without associated TVR is a well-known event. Less is known about the fate of TR after ASD closure and whether a similar late clinical course occurs. However, significant TR late after adult age operation was noticed (some even reported isolated tricuspid surgery in patients with previous ASD closure)^[20]^. It was also shown that TVR associated with ASD closure increased freedom from significant TR late after operation^[13]^. In a heterogeneous adult congenital heart disease group, moderate or greater TR was associated with increased long-term risks (death, heart transplant, or reoperation)^[21]^. Nonetheless, patients with severe TR were proven to have exercise intolerance^[22]^, and even mild TR late after ASD closure was associated with a reduced functional capacity^[23]^.

Corroborating all of this evidence from the literature with our results, it is reasonable to believe that in an adult ASD, one should aim at leaving the operating room with a TR < 2, which means performing TVR in all cases with severe TR and probably also in patients with moderate TR having risk factors for postoperative persistence of TR (age ≥ 60 years, PASP > 50 mmHg, TAD > 40 mm, and tricuspid septal leaflet angle > 30°).

### Limitations

Limitations of our study are related to its retrospective nature and to the lack of follow-up.

## CONCLUSION

In the era of percutaneous device closure, surgery for adult ASD/PAPVC is decreasing in volume, but the proportion of more severe cases, with significant TR, is in net increase (exceeding 50%). Patients showed some decrease in TR after isolated closure of ASD (36±26% of the initial TR grade), but results were significantly better in cases with associated TV surgery (79±23% decrease of TR). We plead for systematic TVR in severe TR associated with supraventricular shunts, and for considering TVR in moderate TR with risk factors of persistence. Large follow-up studies on this matter are still required. Until proven otherwise, extending the aortic cross-clamping time by 10-15 minutes is a small price to pay for the benefit of a long-standing competence of the TV.

**Table:** Authors’ Roles & Responsibilities

AGI	Substantial contributions to the conception and design of the work; and the acquisition, analysis and interpretation of data for the work; drafting the work and revising it critically for important intellectual content; final approval of the version to be published
AP	Substantial contributions to the acquisition, analysis and interpretation of data for the work; drafting the work and revising it; final approval of the version to be published
TAI	Substantial contributions to the acquisition, analysis and interpretation of data for the work; drafting the work and revising it; final approval of the version to be published
ATT	Substantial contributions to the acquisition, analysis and interpretation of data for the work; drafting the work and revising it; final approval of the version to be published
SM	Substantial contributions to the conception of the work; drafting the work and revising it critically; final approval of the version to be published
VAI	Substantial contributions to the conception and design of the work; revising it critically for important intellectual content; final approval of the version to be published
